# Longitudinal enumeration and cluster evaluation of circulating tumor cells improve prognostication for patients with newly diagnosed metastatic breast cancer in a prospective observational trial

**DOI:** 10.1186/s13058-018-0976-0

**Published:** 2018-06-08

**Authors:** Anna-Maria Larsson, Sara Jansson, Pär-Ola Bendahl, Charlotte Levin Tykjaer Jörgensen, Niklas Loman, Cecilia Graffman, Lotta Lundgren, Kristina Aaltonen, Lisa Rydén

**Affiliations:** 1 Department of Clinical Sciences Lund, Division of Oncology and Pathology, Lund University, Lund, Sweden; 2 Department of Hematology, Oncology and Radiation Physics, Skåne University Hospital, Lund, Sweden; 3 Department of Clinical Sciences Lund, Division of Surgery, Lund University, Medicon Village, SE-223 81 Lund, Sweden; 4 Department of Surgery and Gastroenterology, Skåne University Hospital, Malmö, Sweden

**Keywords:** Metastatic breast cancer, Circulating tumor cells (CTCs), Enumeration, Cluster, Prognosis

## Abstract

**Background:**

Circulating tumor cells (CTCs) carry independent prognostic information in patients with metastatic breast cancer (MBC) on different lines of therapy. Moreover, CTC clusters are suggested to add prognostic information to CTC enumeration alone but their significance is unknown in patients with newly diagnosed MBC. We aimed to evaluate whether longitudinal enumeration of circulating tumor cells (CTCs) and CTC clusters could improve prognostication and monitoring of patients with metastatic breast cancer (MBC) starting first-line therapy.

**Methods:**

This prospective study included 156 women with newly diagnosed MBC. CTCs and CTC clusters were detected using CellSearch technology at baseline (BL) and after 1, 3, and 6 months of systemic therapy. The primary end point was progression-free survival (PFS) and the secondary end point overall survival (OS). Median follow-up time was 25 (7–69) months.

**Results:**

There were 79 (52%) and 30 (20%) patients with ≥ 5 CTCs and ≥ 1 CTC cluster at baseline, respectively; both factors were significantly associated with impaired survival. Landmark analyses based on follow-up measurements revealed increasing prognostic hazard ratios for ≥ 5 CTCs and CTC clusters during treatment, predicting worse PFS and OS. Both factors added value to a prognostic model based on clinicopathological variables at all time points and ≥ 5 CTCs and presence of CTC clusters enhanced the model’s C-index to > 0.80 at 1, 3, and 6 months. Importantly, changes in CTCs during treatment were significantly correlated with survival and patients with a decline from ≥ 5 CTCs at BL to < 5 CTCs at 1 month had a similar odds ratio for progression to patients with < 5 CTCs at BL and 1 month. Stratification of patients based on CTC count and CTC clusters into four groups (0 CTCs, 1–4 CTCs, ≥ 5 CTCs, and ≥ 1 CTC + CTC clusters) demonstrated that patients with CTC clusters had significantly worse survival compared to patients without clusters.

**Conclusions:**

Longitudinal evaluation of CTC and CTC clusters improves prognostication and monitoring in patients with MBC starting first-line systemic therapy. The prognostic value increases over time, suggesting that changes in CTC count are clinically relevant. The presence of CTC clusters adds significant prognostic value to CTC enumeration alone.

**Trial registration:**

NCT01322893. Registered on 25 March 2011.

**Electronic supplementary material:**

The online version of this article (10.1186/s13058-018-0976-0) contains supplementary material, which is available to authorized users.

## Background

The prognostic value of circulating tumor cell (CTC) enumeration was first shown in patients with metastatic breast cancer (MBC) assessed by the CellSearch system in 2004 [1]. Since then, several studies have been published in support of these results [2–14] and in 2014 a pooled analysis of data from 1944 patients confirmed that a CTC count of ≥ 5 cells per 7.5 mL blood is an independent predictor of worse progression-free survival (PFS) and overall survival (OS) in patients with MBC [15]. The authors of the pooled analysis developed a clinicopathological prognostication model that included CTC count in addition to other clinically relevant variables, and concluded that CTC-based survival prognostication models should be considered as optimum prognostic models for counselling of patients [15]. Recently, a meta-analysis demonstrated that CTC status can be applied in monitoring the effectiveness of systemic therapy for MBC, since a shift in CTC status between two time points was prognostic [16]. Thus far, most individual studies evaluating CTC count in MBC included patients regardless of prior lines of systemic therapy and baseline CTC was measured in a heterogeneous population of patients on different lines of treatment. The focus of these studies has primarily been on CTC evaluation before starting a new line of treatment or at the first post-treatment evaluation, but no studies have conclusively evaluated long-term monitoring of CTC dynamics. Hence, the presence and dynamics of CTCs during first-line systemic treatment in patients with MBC and its clinical relevance have yet to be fully elucidated. Furthermore, recent studies have shown that detection of CTC clusters in patients with MBC adds prognostic value to CTC enumeration alone [12, 13, 17], but limited data are available on the prognostic value of CTC clusters in previously untreated patients with MBC before and during treatment [18].

The aim of this prospective study was to evaluate longitudinal CTC count ≥ 5 cells/7.5 mL blood and CTC clusters using the CellSearch system as a prognostic instrument in women with newly diagnosed MBC from baseline to 6 months, and examine how these relate to progression-free survival (PFS) and overall survival (OS). A secondary aim was to evaluate if early changes in CTC status can predict response at the first radiological evaluation at 3 months.

## Methods

### Patients and study design

Patients diagnosed with MBC and scheduled for first-line systemic treatment at Skåne University Hospital and Halmstad County Hospital, Sweden, were enrolled into a prospective monitoring trial (ClinicalTrials.gov NCT01322893) conducted by the Department of Oncology and Pathology at Lund University, Sweden. The study was approved by the Lund University Ethics Committee (LU 2010/135). Inclusion criteria were age ≥ 18 years, Eastern Cooperative Oncology Group (ECOG) performance status score 0–2, and predicted life expectancy of > 2 months. Exclusion criteria were prior systemic therapy for metastatic disease, inability to understand the study information, and other malignant disease in the preceding 5 years. After selection, the participating patients started first-line systemic therapy for MBC according to national guidelines; the treating physicians were blind to the CTC results. Patients underwent structured clinical and radiological evaluation every 3 months or at the discretion of the treating physician. Progression versus non-progression was defined according to clinical practice based on clinical and radiological evaluation using the modified Response Evaluation Criteria In Solid Tumors (RECIST) 1.1 [19]. Using this approach progression was defined as progressive disease (PD), whilst non-progression was defined as stable disease (SD), partial response (PR) or complete response (CR).

Samples of whole blood and serum were collected at baseline and after approximately 1, 3, and 6 months of treatment. The serum marker CA15-3 was analyzed at the Department of Clinical Chemistry at Skåne University Hospital with an accredited method used in clinical practice (CA15-3 on Cobas, NPU01449). Twenty-three of the participating patients experienced treatment failure within 6 months of commencement; therefore, they were started on second-line therapy, for which blood sampling was repeated (at baseline after treatment failure (baseline 2) and after 1, 3, and 6 months).

### Detection of CTCs and CTC clusters

Blood samples were collected in 10 mL CellSave Preservation tubes (Menarini Silicon Biosystems, Bologna, Italy), stored between 15 and 30 °C and processed within 96 h of collection. CTCs were isolated and enumerated using the Food and Drug Administration (FDA)-approved CellSearch system (Menarini Silicon Biosystems) as has been described in detail previously [1, 20]. Two investigators certified in the CellSearch technology independently evaluated all images within the generated galleries for events. Any event for which the assessment differed between the investigators was re-evaluated until consensus was reached.

CTC clusters were defined as groups consisting of ≥ 2 CTCs clustered together and with non-overlapping nuclei. Presence of other cell types in addition to CTCs was not documented. Two independent assessors evaluated CTC clusters in CTC galleries exported from the CellTracks Analyzer II system, as described previously [17]. No additional staining of CTCs was performed after the CellSearch analysis was completed. CTC enumeration and CTC clusters were evaluated at baseline and during treatment at 1, 3, and 6 months. A blood sample was considered positive for CTC clusters if ≥ 1 CTC cluster was detected.

### Statistical analysis

Statistical power calculations based on estimated PFS, fraction of patients with a CTC count above the predefined threshold (≥ 5 CTCs), the inclusion period, and the estimated follow-up time determined the required study sample size to be 154 patients (Additional file 1). An additional threshold of ≥ 20 CTCs proposed by Botteri et al. [21] was applied to explore the relationship between the number of CTCs and the presence of CTC clusters.

Categorical or categorized characteristics of the patients, tumors, and CTCs at different time points were compared using Pearson’s chi-squared test or if counts were lower than expected in one or more of the cells, Fisher’s exact test was used. Ordinal data were compared using Pearson’s chi-squared test for trend and variables measured on a continuous scale by the Mann-Whitney U test or, if there were more than two categories, the Kruskal Wallis test.

The primary end point was PFS and the secondary end points were OS and progression versus non-progression at first evaluation, in relation to changes in numbers of CTCs and/or presence of CTC clusters. The study was in accordance with the Reporting Recommendations for Tumor Marker (REMARK) criteria [22]. Time from the date of the blood draw to progression or death from any cause was calculated. If an outcome was not reached the time variables were censored at the last follow up. Kaplan-Meier plots and the log-rank test were used to illustrate and compare survival between subgroups. Survival analysis of variables measured at 1, 3, and 6 months was performed by landmark analysis. Univariable and multivariable hazard ratios (HRs) for selected potential predictors of PFS and OS were determined by Cox proportional hazards regression. Proportional hazards assumptions were checked graphically. Model fit was measured using Harrell’s C-index, and the fit of nested prognostic models was compared using likelihood ratio (LR) tests.

In addition, Cox models with time-dependent covariates were used to estimate the effects of the longitudinally measured binary variables ≥ 5 CTCs and CTC clusters on OS. Briefly, the follow up of each patient was split into multiple non-overlapping episodes for which each of the two covariates were constant. The number of such episodes per patient varied between 1 and 6: 1 episode was sufficient for patients with the same CTC and CTC-cluster status at BL and all follow-up visits. With three follow-up visits (1, 3, and 6 months), change in one or both of these variables can be observed up to three times for patients staying on first-line treatment. Patients who switched to second-line treatment within 6 months from BL can have up to three additional episodes with change in one or both variables. Missing values at follow-up visits were imputed using the principle of last observation carried forward. For example, the HR for ≥ 5 CTCs in models of this kind should be interpreted as the ratio of the mortality during episodes with ≥ 5 CTCs to that of episodes with < 5 CTCs. The method of last observation carried forward is reportedly less prone to selection bias than deletion by list [23], but it is not state of the art within the field of imputation. However, in the light of the low fraction of missing data and the exploratory nature of these analyses, we judge the method reliable.

The association between CTC count and the outcome of the first evaluation was assessed by logistic regression. The value added of CTC count and CTC clusters to a prognostic clinicopathological model was evaluated using LR statistics in Cox regression models, based on a model previously described by Bidard et al. [15]. *P* values in the exploratory analyses were not adjusted for multiple testing and should therefore not be compared to the 5% cutoff. Statistical analysis was with IBM SPSS Statistics (version 24.0, IBM, Armonk, NY, USA) and STATA (version 15.0, StataCorp. College Station, TX, USA).

## Results

### Patient characteristics

In total, 156 patients with newly diagnosed MBC were enrolled in the study between April 2011 and June 2016. There were 31 patients with stage IV disease at initial diagnosis and 125 patients were diagnosed with distant recurrence. Patient and tumor characteristics are summarized in Table 1. The median follow-up time from baseline was 25 months (range 7–69) for patients alive at the last medical visit before the cutoff date of 31 May 2017. The median age at MBC diagnosis was 65 years (range 40–90) and the median metastasis-free interval for patients with recurrent disease was 5.8 years (range 0.4–36.3). Breast cancer subtype was determined in metastases in 114 patients and in primary tumors in 126 patients. There were 105 patients (70%) with estrogen receptor-positive (ER+) tumors, 20 (13%) had human epidermal growth factor receptor 2 positive (HER2+) tumors, and 26 (17%) had triple-negative breast cancer (TNBC), determined primarily from metastatic data, and secondarily from primary tumor data. Visceral metastases (defined as lung, liver, brain, peritoneal, and/or pleural involvement) were present in 91 patients (58%): 36 patients (23%) had bone metastasis only. First-line systemic therapy included endocrine treatment in 58 patients (40%), chemotherapy in 71 patients (49%) and HER2-directed agents in combination with chemotherapy or endocrine therapy in 15 patients (10%).

**Table 1:** Baseline patient and tumor characteristics stratified by CTC count and CTC clusters

	All patients	Baseline CTC < 5	Baseline CTC ≥5	*P* value	Baseline clusters absent	Baseline clusters ≥ 1	*P* value
	(*n* = 156)	(*n* = 73)	(*n* = 79)		(*n* = 122)	(*n* = 30)	
Age MBC, median (range)	65 (40–90)	65 (40–-84)	65 (41–90)	0.71^a^	67 (40–90)	60 (42–72)	0.002^a^
Baseline ECOG							
0	91	48 (53)	43 (47)	0.07^b^	76 (84)	15 (16)	0.32^b^
1	37	17 (46)	20 (54)		29 (78)	8 (22)	
2	22	6 (30)	14 (70)		15 (75)	5 (25)	
Unknown	6						
PT NHG							
I	13	9 (69)	4 (31)	0.58^b^	12 (92)	1 (8)	0.85^b^
II	65	26 (41)	38 (59)		47 (73)	17 (27)	
III	46	22 (49)	23 (51)		38 (84)	7 (16)	
Unknown	32						
PT tumor size							
T1	57	30 (55)	25 (45)	0.16^b^	49 (89)	6 (11)	0.07^b^
T2	51	25 (49)	26 (51)		39 (76)	12 (24)	
T3	20	8 (40)	12 (60)		15 (75)	5 (25)	
T4	19	7 (39)	11 (61)		13 (72)	5 (28)	
Unknown	9						
PT node status							
Negative	44	27 (61)	17 (39)	0.04^c^	39 (89)	5 (11)	0.10^c^
Positive	92	38 (42)	52 (58)		69 (77)	21 (23)	
Unknown	20						
Breast cancer subtype^d^							
ER+ HER2-	105	46 (44)	58 (56)	0.52^c^	86 (83)	18 (17)	0.34^c^
HER2+	20	11 (58)	8 (42)		14 (74)	5 (26)	
ER- HER2-	26	12 (50)	12 (50)		17 (71)	7 (29)	
Unknown	5						
Metastasis-free interval (years)							
0	31	14 (47)	16 (53)	0.57^b^	24 (80)	6 (20)	0.97^b^
> 0-3	28	11 (41)	16 (59)		22 (81)	5 (19)	
> 3	97	48 (51)	47 (49)		76 (80)	19 (20)	
Metastatic sites, number							
< 3	109	58 (54)	49 (46)	0.02^c^	88 (82)	19 (18)	0.34^c^
≥ 3	47	15 (33)	30 (67)		34 (76)	11 (24)	
Site of metastasis							
Non-visceral	65	29 (45)	35 (55)	0.57^c^	47 (73)	17 (27)	0.07^c^
Visceral^e^	91	44 (50)	44 (50)		75 (85)	13 (15)	
1st line treatment for MBC^f^							
Endocrine	58	31 (53)	27 (47)	0.28^c^	56 (97)	2 (3)	< 0.001^c^
Chemotherapy	71	29 (42)	40 (58)		48 (70)	21 (30)	
HER2-targeted	15	9 (60)	6 (40)		11 (73)	4 (27)	
One or more clusters of ≥ 2 CTCs at baseline^g^							
No	122	73 (60)	49 (40)	< 0.001^c^			
Yes	30	0	30 (100)				

*Abbreviations: CTC* circulating tumor cell, *MBC* metastatic breast cancer, *ECOG* Eastern Cooperative Oncology Group, *NHG* Nottingham histological grade, *PT* primary tumor, *ER* estrogen receptor, *HER2* human epidermal growth factor receptor 2

^a^*P* value from Mann-Whitney test

^b^*P* value from Pearson’s chi-squared test for trend

^c^*P* value from Pearson’s chi-squared test

^d^Breast cancer subtype was primarily derived from immunohistochemical staining of the metastasis (*n* = 114). If no information was available from the metastasis, the subtype was derived by staining of the primary tumor (*n* = 36)

^e^Visceral metastasis defined as lung, liver, brain, peritoneal, and/or pleural involvement

^f^A total of 12 patients died and/or treatment was ended before the first structured clinical follow up at 3 months post treatment initiation and consequently no data are available for these patients

^g^Four patients had no baseline sample and thus had no data on CTCs and CTC clusters

### CTC count and CTC clusters

**Fig. 1:**
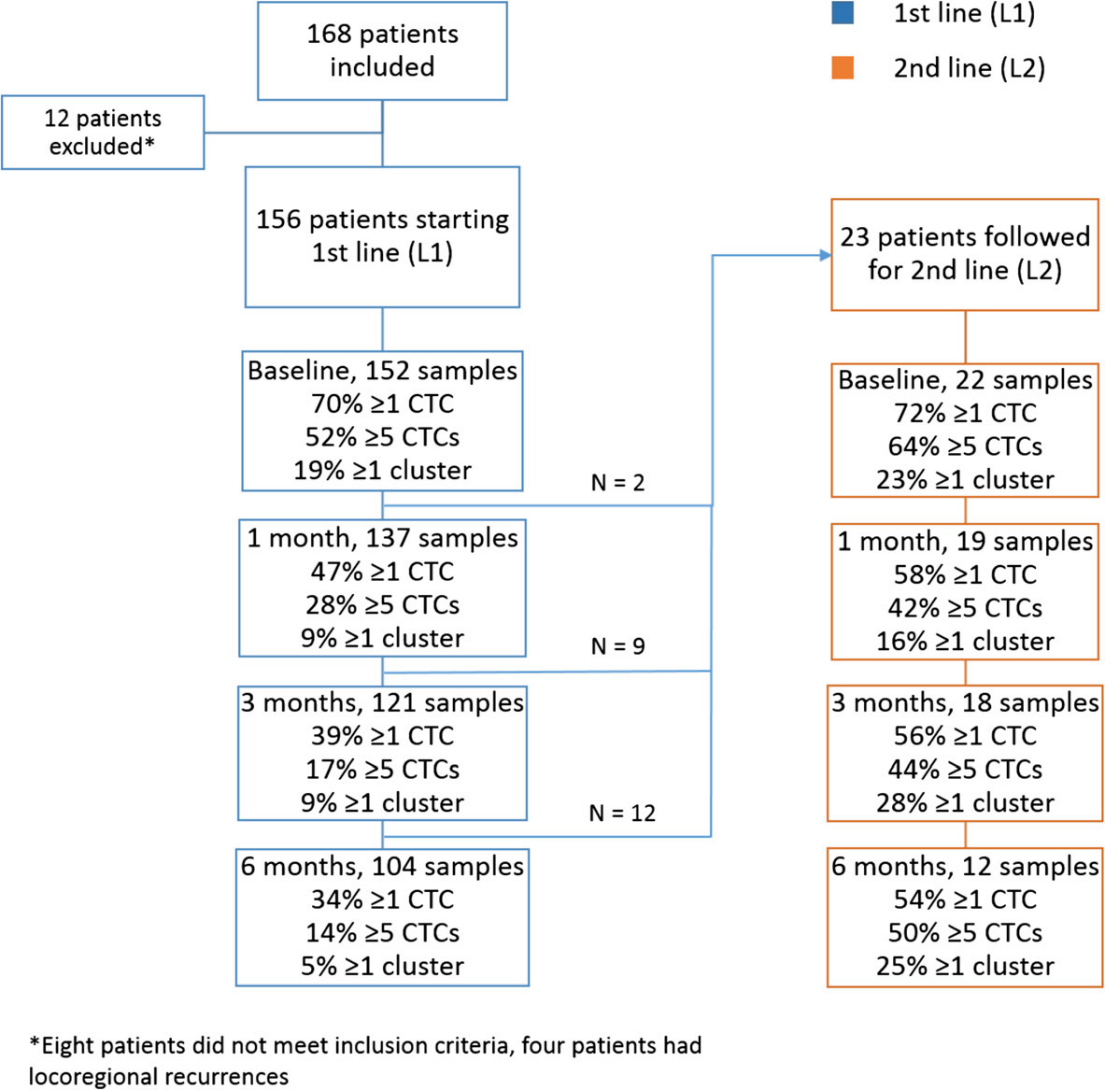
Flowchart of study cohort and time points for circulating tumor cell (CTC) analysis

Blood samples from 115 patients at all time-points (baseline, 1, 3 and 6 months) were analyzed. In total, 591 blood samples were collected and analyzed; two sampling errors and four technical errors were encountered. At baseline, 79 (52%) of 152 evaluable patients had ≥ 5 CTCs (predefined cutoff). The fraction of patients with ≥ 5 CTCs decreased during first-line treatment from baseline to 1, 3, and 6 months, and patients receiving subsequent second-line systemic therapy had on average higher CTC counts at all time points, as depicted in Fig. 1. Applying a cut point of ≥ 20 CTCs, the corresponding numbers for patients with CTCs ≥ 20 were 54/152 (36%), 27/137 (20%), 11/121 (9%), and 8/104 (8%) at baseline, 1, 3, and 6 months, respectively. There were no significant differences in CTC counts between breast cancer subtypes at baseline.

There were 30 patients with CTC clusters at baseline. During first-line systemic therapy, 39 patients had CTC clusters at any time point (baseline, 1, 3 and/or 6 months) and during second-line therapy, 10 patients had CTC clusters (Fig. 1). The presence of CTC clusters was associated with CTC count at all time points and patients with clusters more frequently had ≥ 20 CTCs (Additional file 2). However, there were some patients with clusters and a low CTC count (2–4 CTCs) and half of the patients with a high CTC count (≥ 20 CTCs) did not have any CTC clusters in the sample (Additional file 2). There was no association between breast cancer subtype and presence of CTC clusters.

### Prediction of outcome in relation to CTCs and CTC clusters

**Fig. 2:**
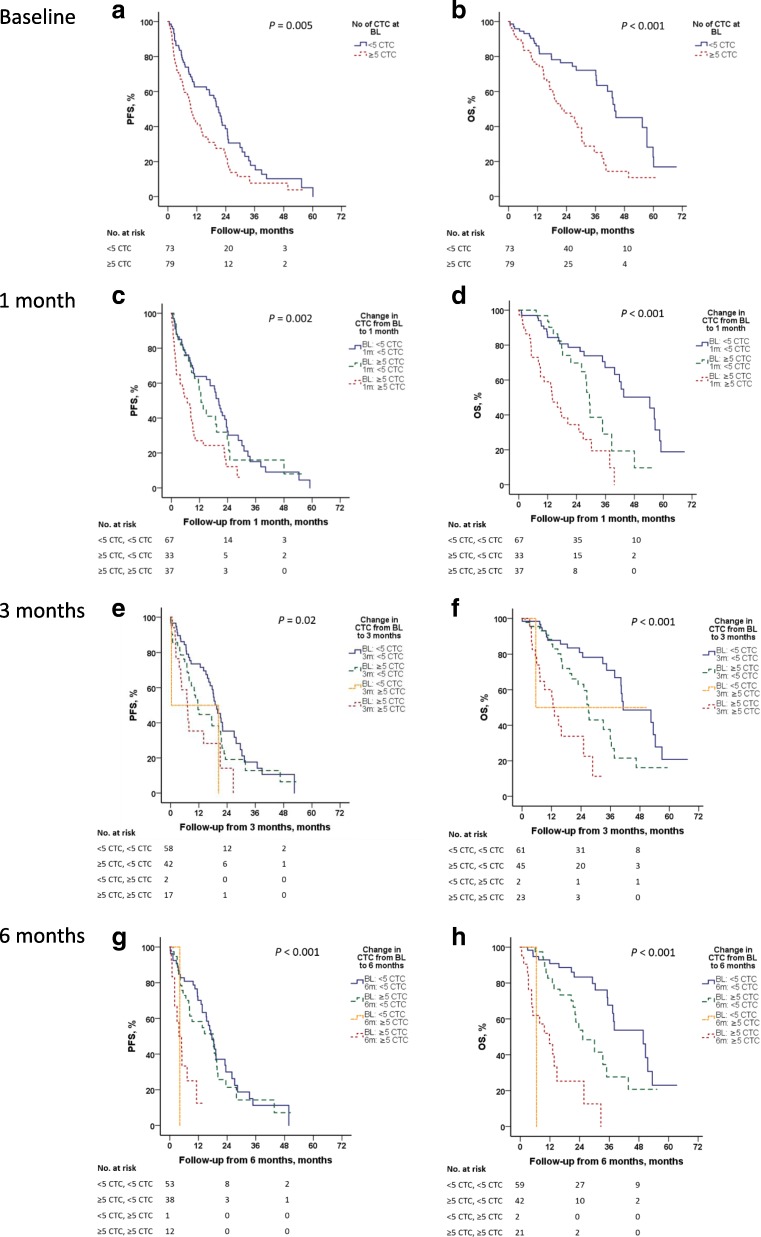
Progression-free survival (PFS) and overall survival (OS) by circulating tumor cell (CTC) count. Kaplan-Meier curves displaying PFS and OS by baseline (BL) CTC count (≥ 5 CTCs) (**a**-**b**), by CTC count at BL and 1 month (**c**-**d**), by CTC count at BL and 3 months (**e**-**f**) and by CTC count at BL and 6 months (**g-h**) during the first 6 months of systemic therapy for MBC. Analyses at 1, 3, and 6 months were performed using landmark analysis, in which the follow-up time was recalculated with a new starting date from the 1, 3, and 6-month sample, respectively

Patients with ≥ 5 CTCs at baseline had inferior PFS (HR_PFS_ 1.68; 95% confidence interval (CI) 1.17–2.42; *P* = 0.005) and OS (HR_OS_ 2.52; 95% CI 1.58–4.01; *P* < 0.001) (Fig. 2a-b). These results remained significant in multivariable analysis even when adjusting for other prognostic factors (Table 2).

**Table 2:** Cox regression hazard ratios for CTC count ≥ 5 versus < 5, and presence versus absence of CTC clusters

	PFS	*P* value	OS	*P* value
	HR (95% CI)		HR (95% CI)	
Baseline				
Unadjusted				
CTC ≥ 5	1.68 (1.17–2.42)	0.005	2.52 (1.58–4.01)	< 0.001
Clusters present	1.54 (1.00–2.40)	0.05	2.23 (1.35–3.69)	0.002
Adjusted^a^				
CTC ≥ 5	2.30 (1.43–3.71)	0.001	3.92 (2.09–7.36)	< 0.001
Clusters present	2.64 (1.46–4.78)	0.001	4.07 (1.99–8.31)	< 0.001
One month^b^				
Unadjusted				
CTC ≥ 5	2.17 (1.43–3.30)	< 0.001	4.38 (2.63–7.30)	< 0.001
Clusters present	3.23 (1.70–-6.14)	< 0.001	4.52 (2.24–9.15)	< 0.001
Adjusted^c^				
CTC ≥ 5	2.30 (1.23–4.32)	0.009	4.39 (2.04–9.43)	< 0.001
Clusters present	3.37 (1.51–7.55)	0.003	5.67 (2.30–13.95)	< 0.001
Three months^b^				
Unadjusted				
CTC ≥ 5	2.24 (1.24–4.03)	0.07	3.28 (1.76–6.12)	< 0.001
Clusters present	3.16 (1.60–6.22)	0.001	3.35 (1.68–6.68)	0.001
Adjusted^c^				
CTC ≥ 5	2.95 (1.44–6.06)	0.003	5.93 (2.62–13.42)	< 0.001
Clusters present	3.04 (1.35–6.84)	0.007	3.55 (1.44–8.77)	0.006
Six months^b^				
Unadjusted				
CTC ≥ 5	4.33 (2.10–8.93)	< 0.001	7.74 (3.52–16.99)	< 0.001
Clusters present	6.48 (2.26–18.56)	0.001	9.92 (3.30–29.78)	< 0.001
Adjusted^c^				
CTC ≥ 5	6.43 (2.30–17.94)	< 0.001	15.72 (3.79–65.17)	< 0.001
Clusters present	7.17 (2.03–25.36)	0.002	21.65 (5.06–92.63)	< 0.001

*Abbreviations: PFS* progression-free survival, *OS* overall survival, *HR* hazard ratio, *CTC* circulating tumor cell

^a^Adjusted for the variables included in the clinicopathological model (Additional file 3)

^b^Assessed by landmark analysis

^c^Adjusted for the variables included in the clinicopathological model (Additional file 3) and for baseline CTC count (< 5 vs ≥ 5)

HRs increased time-dependently during treatment in longitudinal landmark analysis of CTC count, predicting worse PFS and OS from all follow-up sample time points in patients with CTCs ≥ 5 in the sample (Table 2). A reduction in CTC count during systemic therapy from ≥ 5 CTCs at baseline to < 5 CTCs at follow up (at 1, 3, and 6 months) was also significantly associated with improved survival, in comparison to patients with persistent CTCs ≥ 5 at 1, 3, and 6 months (Fig. 2c-h). Univariable Cox regression analysis of OS with time-varying covariates confirmed the poor prognosis in patients with high CTC count, and the mortality in patients with CTCs ≥ 5 was 5.74 (95% CI 3.48–9.48) times higher than in those with CTCs < 5. The corresponding mortality ratio after adjustment for clinicopathological variables (listed in Additional file 3) was 9.01 (95% CI 4.70–17.2). When applying the threshold of ≥ 20 CTCs in relation to outcome (Additional file 4), the HRs for progression or death were higher at all time points compared to analysis with the predefined cutoff of ≥ 5 CTCs, shown in Table 2.

Patients with presence of ≥ 1 CTC cluster at baseline had inferior OS and PFS compared to patients without CTC clusters (Table 2). HRs for presence versus absence of CTC clusters increased during systemic treatment for both PFS and OS (Table 2). For CTC count, on Cox regression with time-dependent covariates there was significantly higher mortality in episodes with CTC clusters compared to episodes without CTC clusters (univariable HR 5.14; 95% CI 2.86–9.24; multivariable HR 6.23; 95% CI 3.56–13.50). In a bivariate Cox model including CTCs ≥ 5 and CTC clusters, mortality was 7.79 times higher during episodes where both factors were unfavorable compared to episodes where both factors were favorable. The corresponding HR for mortality was 11.5 in a model adjusted for clinicopathological variables.

**Fig. 3:**
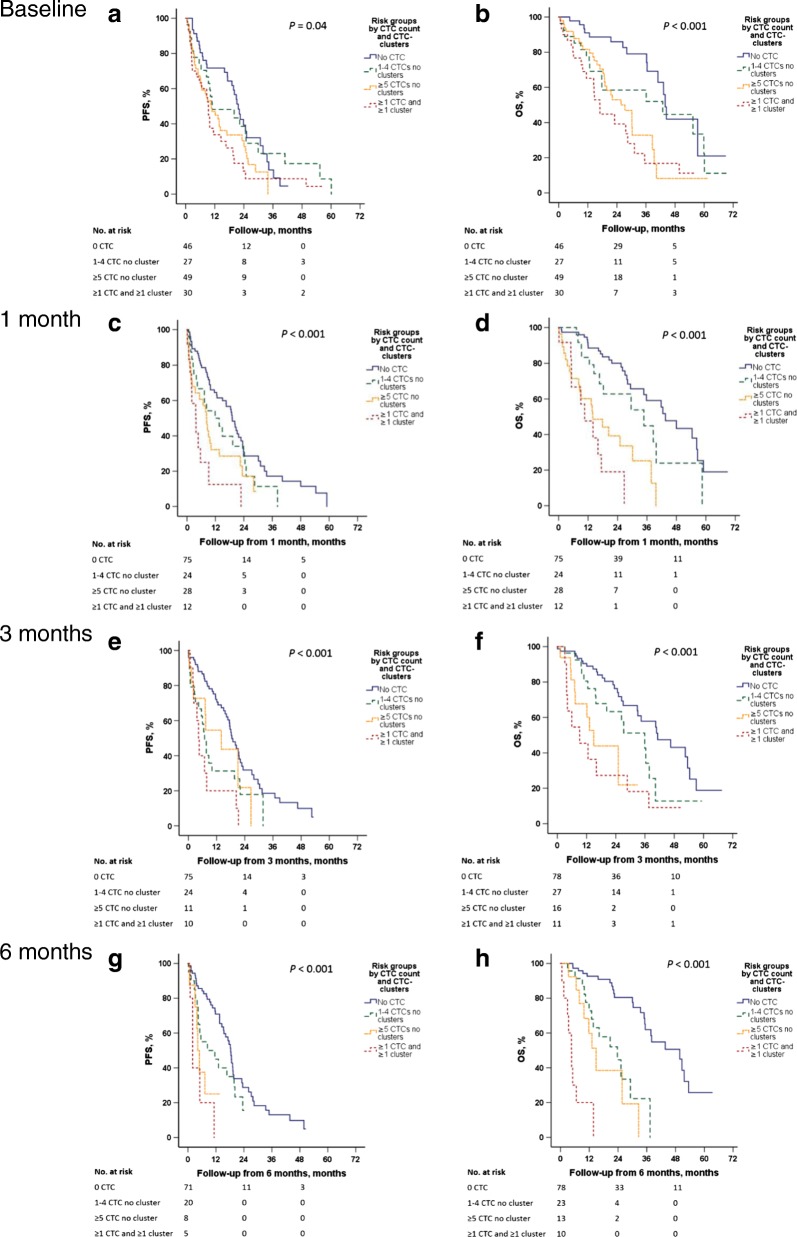
Progression-free survival (PFS) and overall survival (OS) by circulating tumor cell (CTC) count and CTC cluster detection. Kaplan-Meier curves displaying PFS and OS by four groups including CTC count and CTC cluster detection at baseline (**a**-**b**) at 1 month (**c**-**d**), 3 months (**e**-**f**) and 6 months (**g**-**h**). The four groups were patients with no CTCs, patients with 1–4 CTCs and no clusters, patients with ≥ 5 CTCs and no clusters, and patients with > 1 CTC and clusters. Analyses at 1, 3, and 6 months were performed with landmark analysis where the follow-up time was recalculated with a new starting date from the 1, 3, and 6-month samples, respectively

Stratifying patients based on CTC count and presence of CTC clusters revealed four risk groups (0 CTC; 1–4 CTCs, 0 clusters; ≥ 5 CTCs, 0 clusters; and ≥ 1 CTC, ≥1 cluster) where patients with CTC clusters had the worst PFS and OS from all evaluated time points (Fig. 3a-h). When applying the cutoff of ≥ 20 CTCs, presence of CTC clusters was no longer significantly prognostic (data not shown).

To evaluate CTCs as an early predictor of progression in MBC, changes in CTC count from baseline to 1 month or 3 months were analyzed in relation to the outcome of the first evaluation at 3 months (progression versus non-progression). In logistic regression models, patients with a rapid decrease in CTC count (from baseline to 1 month) had an odds of progression that did not differ significantly compared to patients with a consistently low (< 5) CTC count (odds ratio (OR) 1.27; *P* = 0.7, Additional file 5), supporting the notion that a rapid decrease in CTCs is important. In contrast, patients with a high CTC count at 1 month, in addition to those with a high CTC count at 3 months, had significantly higher odds of progression at the 3-month evaluation when compared to patients with low CTC count at baseline and at 1 and 3 months (ORs, 4.21 and 9.16, respectively; Additional file 5). Furthermore, compared to the reference group with persistent low CTC count at baseline and 3 months, patients with a decrease in CTCs from baseline to 3 months had 3.56 times higher odds of progression (9 patients with progression and 36 with non-progressions compared to 4 with progression and 57 with non-progression; OR = (9/36)/(4/57); Additional file 5).

### Prognostication by a clinicopathological model including CTC count and CTC clusters

To evaluate the value added by CTC count and CTC clusters compared to the currently used clinical prognostic variables, we built a prognostic model based on the previously published model by Bidard et al. [15]. The baseline model included clinicopathological variables reported by Bidard et al. to have significant prognostic value in pooled analysis and included breast cancer subtype, histologic grade, ECOG performance status, age, metastasis-free interval, and visceral metastases (Additional file 3). In addition, the number of metastatic locations was included in our model since this was a significant prognostic marker in univariable analysis in the present cohort. The commonly used serum marker CA15-3 did not show prognostic value in our univariable analysis and was therefore not included in the model (Additional file 6).

The addition of baseline CTC count (CTC_BL_, cutoff ≥ 5 CTCs) and baseline CTC clusters (CTC cluster_BL_, cutoff ≥ 1 cluster of ≥ 2 CTCs) to the baseline clinicopathological model constructed in this cohort, revealed a significant improvement in survival prognostication for both PFS and OS when the variables were added separately (Table 3). Follow-up samples from 1, 3, and 6 months were also evaluated in the prognostic model to assess the value added by CTC count and detection of CTC clusters during treatment. This demonstrated that both CTC count ≥ 5 cells and presence of CTC clusters improved the model at all time points and onwards for both PFS and OS. Notably, the improvement in prognostication was stronger for OS from all follow-up time points (compared to baseline) where CTCs and CTC cluster presence enhanced the model C-index to > 0.80 at 3 and 6 months (Table 3).

**Table 3:** Prognostic information of CTC count and CTC clusters in a clinicopathological model

Model 1	Model 1 C-index	Model 2	Model 2 C-index	LRχ^2^	df	*P* value
PFS at baseline^a^						
CP	0.690	CP + CTC_BL_	0.707	11.46	1	0.0007
CP	0.690	CP + cluster	0.706	9.47	1	0.0021
CP	0.690	CP + CTC_BL_ + cluster	0.714	14.46	2	0.0007
OS at baseline^a^						
CP	0.752	CP + CTC_BL_	0.786	18.96	1	< 0.0001
CP	0.752	CP + cluster_BL_	0.777	13.16	1	0.0003
CP	0.752	CP + CTC_BL_ + cluster_BL_	0.799	23.54	2	< 0.0001
PFS at 1 month^b^						
CP + CTC_BL_	0.697	CP + CTC_BL_ + CTC_1M_	0.709	6.69	1	0.0097
CP + CTC_BL_	0.697	CP + CTC_BL_ + cluster_1M_	0.712	7.56	1	0.0060
CP + CTC_BL_	0.697	CP + CTC_BL_ + CTC_1M+_cluster_1M_	0.713	10.56	2	0.0051
OS at 1 month^b^						
CP + CTC_BL_	0.766	CP + CTC_BL_ + CTC_1M_	0.812	15.73	1	0.0001
CP + CTC_BL_	0.766	CP + CTC_BL_ + cluster_1M_	0.788	12.01	1	0.0005
CP + CTC_BL_	0.766	CP + CTC_BL_ + CTC_1M+_cluster_1M_	0.817	20.57	2	< 0.0001
PFS at 3 months^b^						
CP + CTC_BL_	0.695	CP + CTC_BL_ + CTC_3M_	0.701	7.31	1	0.0068
CP + CTC_BL_	0.695	CP + CTC_BL_ + cluster_3M_	0.711	6.22	1	0.0126
CP + CTC_BL_	0.695	CP + CTC_BL_ + CTC_3M+_cluster_3M_	0.710	9.01	2	0.0110
OS at 3 months^b^						
CP + CTC_BL_	0.774	CP + CTC_BL_ + CTC_3M_	0.806	14.76	1	0.0001
CP + CTC_BL_	0.774	CP + CTC_BL_ + cluster_3M_	0.806	7.02	1	0.0081
CP + CTC_BL_	0.774	CP + CTC_BL_ + CTC_3M+_cluster_3M_	0.806	15.16	2	0.0005
PFS at 6 months^b^						
CP + CTC_BL_	0.694	CP + CTC_BL_ + CTC_6M_	0.732	11.14	1	0.0008
CP + CTC_BL_	0.694	CP + CTC_BL_ + cluster_6M_	0.709	7.14	1	0.0075
CP + CTC_BL_	0.694	CP + CTC_BL_ + CTC_6M+_cluster_6M_	0.727	12.14	2	0.0023
OS at 6 months^b^						
CP + CTC_BL_	0.758	CP + CTC_BL_ + CTC_6M_	0.804	15.88	1	0.0001
CP + CTC_BL_	0.758	CP + CTC_BL_ + cluster_6M_	0.813	13.24	1	0.0003
CP + CTC_BL_	0.758	CP + CTC_BL_ + CTC_6M+_cluster_6M_	0.818	20.66	2	< 0.0001

*Abbreviations: BL* baseline, *3M* 3 months, *6M* 6 months, *df* degrees of freedom, *LRχ*^*2*^ likelihood ratio chi-square, *CP* clinicopathological model, *CTC* circulating tumor cell, *PFS* progression-free survival, *OS* overall survival

^a^Adjusted for subtype, histologic grade, performance status (Eastern Cooperative Oncology Group (ECOG)), age, metastasis-free interval (MFI), visceral metastases, and number of metastatic locations

^b^Adjusted for baseline CTC count ≥ 5, subtype, histologic grade, performance status (ECOG), age, MFI, visceral metastases, and number of metastatic locations. Analyses at 1, 3, and 6 months were performed by landmark analysis

## Discussion

In this study, we showed that longitudinal evaluation of CTC and CTC cluster dynamics for 6 months improves prognostication and treatment monitoring in patients with MBC who are starting first-line systemic therapy. Elevated CTC count ≥ 5 CTCs and detection of CTC clusters were prognostic from all investigated time points, and independently added significant value to a prognostic clinicopathological model at baseline and during follow up. Importantly, changes in CTC count throughout treatment significantly correlated with survival and the prognostic value was more prominent at later time points. To the best of our knowledge, this is the first study to describe the longitudinal dynamics and independent prognostic value of CTCs and CTC clusters within a prospective cohort of patients newly diagnosed with MBC and starting first-line systemic therapy.

The prognostic value of CTC count in patients with MBC has been confirmed at the highest level of evidence [15] in a pooled analysis; however, previous studies evaluating CTC enumeration in patients with newly diagnosed MBC are sparse and have mainly focused on its prognostic value at baseline [12] or first follow up [9]. Few studies have addressed the value added by detection of CTC clusters [12, 13, 17], and the ones that have, were mostly performed in mixed populations and were not focused on patients starting first-line systemic therapy. Furthermore, the study design was often retrospective [2, 3, 7] and/or they included only patients fulfilling certain pre-specified criteria such as a specific subtype [14] or type of systemic therapy [6].

During first-line systemic therapy in the present study, CTC count decreased rapidly in most patients indicating systemic treatment efficacy. In contrast, patients who switched to second-line systemic therapy more often had ≥ 5 CTCs and did not experience a similar decline in CTC count during treatment (Fig. 1). A change in CTC count from baseline to follow up at 1, 3, and 6 months was prognostic at all time points, and patients with persistent CTCs ≥ 5 had worse PFS and OS compared to patients with ≥ 5 CTCs at baseline but < 5 CTCs in follow-up samples. This is in accordance with previous studies in cohorts that were more heterogeneous [15, 24] and with a recently published meta-analysis reporting that CTC status predicts treatment response in patients with MBC [16]. In addition, these results support findings in the recent SWOG S0500 trial suggesting that patients with persistent CTCs ≥ 5 during systemic treatment may harbor cancers that are more resistant to chemotherapy [11]. However, CTC enumeration alone is not able to elucidate the molecular mechanisms responsible for therapy resistance, nor provide guidance on the selection of systemic therapy. Further molecular characterization of CTCs is important for this purpose, and might provide a basis for modification of future treatment based on CTC molecular subtyping.

The persistent presence of ≥ 5 CTCs at baseline, 1, and 3 months significantly increased the OR for progression at the first-response evaluation. Interestingly, we found that patients who had a decrease in CTCs from ≥ 5 at baseline to < 5 at 1 month had the same probability of progression at first evaluation as those with consistently low CTC count. The usefulness of CTC count in prediction of treatment efficacy has been shown [16, 25, 26], and our results support the application of CTCs as a marker of therapy response in women with MBC receiving systemic therapy. Our results show that CTC enumeration and CTC clusters are promising candidates for evaluation of therapy efficacy in MBC and provide reliable prognostication during follow up.

This study is one of the largest to evaluate CTC clusters in patients with MBC on first-line systemic therapy during long-term follow up. The results convincingly showed that the presence of CTC clusters was significantly and independently prognostic at all investigated time points and could identify patients with worse prognosis than those with ≥ 5 CTCs alone. This is in line with our previous results [17] and other recently published studies showing that CTC clusters add prognostic value to CTC enumeration in women with MBC [12, 13]. Incorporating CTC counts and CTC clusters into the clinicopathologic prognostication model proposed by Bidard et al. revealed that these factors significantly improved prognostication at all time points for both PFS and OS. Cox regression analysis of OS with time-varying covariates showed that mortality was increased for episodes with concomitant presence of CTCs ≥ 5 and CTC clusters (HRs, 5.7 and 5.1, respectively), thus confirming the poor prognosis over time in patients with high CTC count and presence of CTC clusters. For patients with both high CTC count (CTCs ≥ 5) and presence of CTC clusters, the mortality was 11 times higher than in patients without these factors when adjusting for standard clinicopathological variables. However, presence of ≥ 20 CTCs was strongly associated with the presence of CTC clusters, and thus CTC clusters did not add any significant prognostic information after adjustment for a high CTC count ≥ 20. Notably, half of patients with ≥ 20 CTCs did not have CTC clusters, and some patients with a CTC count < 5 did have CTC clusters. There were few patients with a high CTC count without clusters and a low CTC count and presence of CTC clusters (Additional file 2), therefore it would not have been meaningful to perform survival analyses for these subgroups. These results further support the use of CTC count and CTC cluster presence to improve prognostication in patients with MBC at baseline and during follow-up.

The presence of CTC clusters added significant value independently of CTC count ≥ 5 CTCs, which underlines the importance of future research focusing on the biological significance of CTC clusters in patients with MBC. Previous studies have shown that CTC clusters have a higher metastatic potential than single CTCs [27–30] and CTC cluster-mediated metastasis has emerged as an alternative model of metastatic seeding along with epithelial-mesenchymal transition (EMT) [18]. Others hypothesize that clusters shed into the circulation as an entity composed of several tumor cells [27] and sometimes with platelets and/or leukocytes. This is in contrast to the EMT model in which single cells enter the bloodstream after transformation into a mesenchymal phenotype. Clearly, the clusters are tumor cells with an improved capacity to survive in the circulation and can avoid clearance by sheer force. Several studies have shown that none or very few CTCs within clusters are apoptotic, whereas a relatively large number of single CTCs are [14, 17, 31]. The widely used CellSearch system easily identifies CTC clusters and thus it would be feasible to assess them in all centers that possess this technology.

Survival analysis was performed including patients with 1–4 CTCs (normally considered low risk and grouped with patients with no CTCs) as a separate group to further explore CTC count and CTC cluster detection. This revealed that 6 months after therapy initiation, patients with 1–4 CTCs had PFS and OS closer to patients with ≥ 5 CTCs than to patients with no CTCs, indicating that even a small number of CTCs in addition to the predefined prognostic cutoff ≥ 5 CTCs [1, 15] might be informative at later follow-up time points. In early breast cancer, a prognostic CTC cutoff ≥ 1 CTC has been proposed [32], and in a metastatic setting this has also been suggested as an alternative cutoff [33]. Furthermore, a threshold of ≥20 CTCs was linked to higher incidence of progression and death at all time points, compared to when the predefined cutoff ≥ 5 CTCs was applied. This study was not powered to evaluate cutoffs other than the predefined cutoff ≥ 5 CTCs and our results support the prognostic value of this cutoff. However, our findings also suggest that the threshold for CTC number needs to be interpreted with care (Fig. 3a-h), particularly during follow up. CTC dynamics over time seem to be essential for estimating prognosis, especially in patients with a reduction in CTCs during treatment for whom the estimated prognosis improves.

A strength of this study is the prospective design of serial CTC and CTC cluster evaluation over 6 months in women with newly diagnosed MBC, including sampling before the start of first-line systemic therapy and structured evaluation at pre-specified intervals. The median follow-up time from baseline was 25 (range 7–69) months and the follow-up data were extensive as few patients terminated the study prematurely. Molecular data to determine breast cancer subtype were available from metastasis biopsies from 73% of patients. Furthermore, the treating physicians were blinded to the CTC results, which avoided treatment bias. This study thus enabled investigation of the presence and dynamics of CTCs and CTC clusters during the first 6 months of treatment, and we applied structured monitoring and blood sampling at predefined time points. A potential weakness of this study is the long inclusion period related to the strict inclusion criteria that included only newly diagnosed cases of MBC before the start of first-line therapy and ECOG performance status score between 0 and 2. Moreover, we included patients irrespective of type of systemic therapy and thus we can draw no conclusion on treatment response related to a specific type of therapy.

## Conclusion

The results of this study support the clinical utility of longitudinal CTC and CTC cluster evaluation for prognostication and treatment monitoring in patients with MBC, who are starting first-line systemic therapy. The prognostic value of CTC count ≥ 5 CTCs and CTC cluster evaluation increased over time, suggesting that the dynamic changes in CTCs and CTC clusters are more relevant to prognosis than a single baseline enumeration. Presence of CTC clusters added significant prognostic value to CTC enumeration alone and standard clinicopathological characteristics at all time points and could identify a subgroup of patients with a notably worse prognosis. These findings are highly relevant for improving prognostication in MBC and in helping clinicians monitor patients with MBC during systemic therapy.

## Additional files

Additional file 1: Supplementary information: power calculation performed before initiation of the study. (PDF 483 kb)

Additional file 2: **Figure S1.** CTC count as a continuous variable in relation to presence of CTC clusters. (PDF 182 kb)

Additional file 3: **Table S1.** Multivariable Cox regression analysis of prognostic variables. (PDF 426 kb)

Additional file 4: **Table S2.** Unadjusted Cox regression HRs for CTC count ≥ 5 vs < 5 CTCs, and CTC count ≥ 20 vs < 20 CTCs. (PDF 114 kb)

Additional file 5: **Table S3.** Change in CTC count in relation to progression versus non-progression at first radiological evaluation. (PDF 131 kb)

Additional file 6: **Table S4.** Unadjusted Cox regression analyses of patient and tumor characteristics at baseline. (PDF 146 kb)

## Abbreviations

BL: Baseline
CI: Confidence interval
CK: Cytokeratin
CR: Complete response
CTC: Circulating tumor cell
ECOG: Eastern Cooperative Oncology Group
EMT: Epithelial-mesenchymal transition
EpCAM: Epithelial cell adhesion molecule
ER: Estrogen receptor
FU: Follow-up
HER2: Human epidermal growth factor receptor 2
HR: Hazard ratio
KM: Kaplan-Meier
LR: Likelihood ratio
MBC: Metastatic breast cancer
OR: Odds ratio
OS: Overall survival
PFS: Progression-free survival
PgR: Progesterone receptor
PR: Partial response
RECIST: Response Evaluation Criteria in Solid Tumors
REMARK: Reporting recommendations for tumor marker
SD: Stable disease
TNBC: Triple-negative breast cancer

## References

[1] Cristofanilli M, Budd GT, Ellis MJ, Stopeck A, Matera J, Miller MC, Reuben JM, Doyle GV, Allard WJ, Terstappen LW (2004). Circulating tumor cells, disease progression, and survival in metastatic breast cancer. N Engl J Med.

[2] Cristofanilli M, Broglio KR, Guarneri V, Jackson S, Fritsche HA, Islam R, Dawood S, Reuben JM, Kau SW, Lara JM (2007). Circulating tumor cells in metastatic breast cancer: biologic staging beyond tumor burden. Clin Breast Cancer.

[3] Dawood S, Broglio K, Valero V, Reuben J, Handy B, Islam R, Jackson S, Hortobagyi GN, Fritsche H, Cristofanilli M (2008). Circulating tumor cells in metastatic breast cancer: from prognostic stratification to modification of the staging system?. Cancer.

[4] Nole F, Munzone E, Zorzino L, Minchella I, Salvatici M, Botteri E, Medici M, Verri E, Adamoli L, Rotmensz N (2008). Variation of circulating tumor cell levels during treatment of metastatic breast cancer: prognostic and therapeutic implications. Ann Oncol.

[5] Nakamura S, Yagata H, Ohno S, Yamaguchi H, Iwata H, Tsunoda N, Ito Y, Tokudome N, Toi M, Kuroi K (2010). Multi-center study evaluating circulating tumor cells as a surrogate for response to treatment and overall survival in metastatic breast cancer. Breast Cancer.

[6] Hartkopf AD, Wagner P, Wallwiener D, Fehm T, Rothmund R (2011). Changing levels of circulating tumor cells in monitoring chemotherapy response in patients with metastatic breast cancer. Anticancer Res.

[7] Giuliano M, Giordano A, Jackson S, Hess KR, De Giorgi U, Mego M, Handy BC, Ueno NT, Alvarez RH, De Laurentiis M (2011). Circulating tumor cells as prognostic and predictive markers in metastatic breast cancer patients receiving first-line systemic treatment. Breast Cancer Res.

[8] Pierga JY, Hajage D, Bachelot T, Delaloge S, Brain E, Campone M, Dieras V, Rolland E, Mignot L, Mathiot C (2012). High independent prognostic and predictive value of circulating tumor cells compared with serum tumor markers in a large prospective trial in first-line chemotherapy for metastatic breast cancer patients. Ann Oncol.

[9] Martin M, Custodio S, de Las Casas ML, Garcia-Saenz JA, de la Torre JC, Bellon-Cano JM, Lopez-Tarruella S, Vidaurreta-Lazaro M, de la Orden V, Jerez Y (2013). Circulating tumor cells following first chemotherapy cycle: an early and strong predictor of outcome in patients with metastatic breast cancer. Oncologist.

[10] Wallwiener M, Riethdorf S, Hartkopf AD, Modugno C, Nees J, Madhavan D, Sprick MR, Schott S, Domschke C, Baccelli I (2014). Serial enumeration of circulating tumor cells predicts treatment response and prognosis in metastatic breast cancer: a prospective study in 393 patients. BMC Cancer.

[11] Smerage JB, Barlow WE, Hortobagyi GN, Winer EP, Leyland-Jones B, Srkalovic G, Tejwani S, Schott AF, O'Rourke MA, Lew DL (2014). Circulating tumor cells and response to chemotherapy in metastatic breast cancer: SWOG S0500. J Clin Oncol.

[12] Mu Z, Wang C, Ye Z, Austin L, Civan J, Hyslop T, Palazzo JP, Jaslow R, Li B, Myers RE (2015). Prospective assessment of the prognostic value of circulating tumor cells and their clusters in patients with advanced-stage breast cancer. Breast Cancer Res Treat.

[13] Wang C, Mu Z, Chervoneva I, Austin L, Ye Z, Rossi G, Palazzo JP, Sun C, Abu-Khalaf M, Myers RE (2017). Longitudinally collected CTCs and CTC-clusters and clinical outcomes of metastatic breast cancer. Breast Cancer Res Treat.

[14] Paoletti C, Li Y, Muniz MC, Kidwell KM, Aung K, Thomas DG, Brown ME, Abramson VG, Irvin WJ, Lin NU (2015). Significance of circulating tumor cells in metastatic triple-negative breast cancer patients within a randomized, phase II trial: TBCRC 019. Clin Cancer Res.

[15] Bidard FC, Peeters DJ, Fehm T, Nole F, Gisbert-Criado R, Mavroudis D, Grisanti S, Generali D, Garcia-Saenz JA, Stebbing J (2014). Clinical validity of circulating tumour cells in patients with metastatic breast cancer: a pooled analysis of individual patient data. Lancet Oncol.

[16] Yan WT, Cui X, Chen Q, Li YF, Cui YH, Wang Y, Jiang J. Circulating tumor cell status monitors the treatment responses in breast cancer patients: a meta-analysis. Sci Rep. 2017;7:43464. 10.1038/srep43464. 10.1038/srep43464 PMC5364512 28337998

[17] Jansson S, Bendahl PO, Larsson AM, Aaltonen KE, Ryden L (2016). Prognostic impact of circulating tumor cell apoptosis and clusters in serial blood samples from patients with metastatic breast cancer in a prospective observational cohort. BMC Cancer.

[18] Fabisiewicz A, Grzybowska E. CTC clusters in cancer progression and metastasis. Med Oncol. 2017;34(1):12. 10.1007/s12032-016-0875-0. 10.1007/s12032-016-0875-0 28012133

[19] Eisenhauer EA, Therasse P, Bogaerts J, Schwartz LH, Sargent D, Ford R, Dancey J, Arbuck S, Gwyther S, Mooney M (2009). New response evaluation criteria in solid tumours: revised RECIST guideline (version 1.1). Eur J Cancer.

[20] Allard WJ, Matera J, Miller MC, Repollet M, Connelly MC, Rao C, Tibbe AG, Uhr JW, Terstappen LW (2004). Tumor cells circulate in the peripheral blood of all major carcinomas but not in healthy subjects or patients with nonmalignant diseases. Clin Cancer Res.

[21] Botteri E, Sandri MT, Bagnardi V, Munzone E, Zorzino L, Rotmensz N, Casadio C, Cassatella MC, Esposito A, Curigliano G (2010). Modeling the relationship between circulating tumour cells number and prognosis of metastatic breast cancer. Breast Cancer Res Treat.

[22] McShane LM, Altman DG, Sauerbrei W, Taube SE, Gion M, Clark GM (2005). Reporting recommendations for tumor marker prognostic studies (REMARK). J Natl Cancer Inst.

[23] Molenberghs G, Kenward MG (2007). Missing data in clinical studies, 1st edition.

[24] Hayes DF, Cristofanilli M, Budd GT, Ellis MJ, Stopeck A, Miller MC, Matera J, Allard WJ, Doyle GV, Terstappen LW (2006). Circulating tumor cells at each follow-up time point during therapy of metastatic breast cancer patients predict progression-free and overall survival. Clin Cancer Res.

[25] Budd GT, Cristofanilli M, Ellis MJ, Stopeck A, Borden E, Miller MC, Matera J, Repollet M, Doyle GV, Terstappen LW (2006). Circulating tumor cells versus imaging−predicting overall survival in metastatic breast cancer. Clin Cancer Res.

[26] Liu MC, Shields PG, Warren RD, Cohen P, Wilkinson M, Ottaviano YL, Rao SB, Eng-Wong J, Seillier-Moiseiwitsch F, Noone AM (2009). Circulating tumor cells: a useful predictor of treatment efficacy in metastatic breast cancer. J Clin Oncol.

[27] Aceto N, Bardia A, Miyamoto DT, Donaldson MC, Wittner BS, Spencer JA (2014). Yu M, Pely A, Engstrom A, Zhu H et al: Circulating tumor cell clusters are oligoclonal precursors of breast cancer metastasis. Cell.

[28] Watanabe S (1954). The metastasizability of tumor cells. Cancer.

[29] Fidler IJ (1973). The relationship of embolic homogeneity, number, size and viability to the incidence of experimental metastasis. Eur J Cancer.

[30] Liotta LA, Saidel MG, Kleinerman J (1976). The significance of hematogenous tumor cell clumps in the metastatic process. Cancer Res.

[31] Hou JM, Krebs MG, Lancashire L, Sloane R, Backen A, Swain RK, Priest LJ, Greystoke A, Zhou C, Morris K (2012). Clinical significance and molecular characteristics of circulating tumor cells and circulating tumor microemboli in patients with small-cell lung cancer. J Clin Oncol.

[32] Rack B, Schindlbeck C, Juckstock J, Andergassen U, Hepp P, Zwingers T, Friedl TW, Lorenz R, Tesch H, Fasching PA, et al. Circulating tumor cells predict survival in early average-to-high risk breast cancer patients. J Natl Cancer Inst. 2014;106(5):1-11. 10.1093/jnci/dju273. 10.1093/jnci/dju066 PMC4112925 24832787

[33] Shiomi-Mouri Y, Kousaka J, Ando T, Tetsuka R, Nakano S, Yoshida M, Fujii K, Akizuki M, Imai T, Fukutomi T (2016). Clinical significance of circulating tumor cells (CTCs) with respect to optimal cut-off value and tumor markers in advanced/metastatic breast cancer. Breast Cancer.

