# Supplementary material for: Longitudinal enumeration and cluster evaluation of circulating tumor cells improve prognostication for patients with newly diagnosed metastatic breast cancer in a prospective observational trial
Source: Breast Cancer Res. 2018 Jun 8;20:48. doi: 10.1186/s13058-018-0976-0 (PMC5994056; doi:10.1186/s13058-018-0976-0)
Supplement: Supplementary file 1 — Supplementary information: power calculation performed before initiation of the study. (PDF 483 kb) [file 13058_2018_976_MOESM1_ESM.pdf]

Supplementary information

Power calculation

The initial power calculation for the study estimated that 144 patients would be sufficient to have 80% power to detect a difference in response rate of the size 30% vs 10%, for two groups (CTC<5 and CTC≥5) of equal size, as significant at the alpha level 0.05 with Fisher´s exact test. But, the plan was to use the more powerful log rank test to test for differences in survival between the two groups. Hence the power would most likely be higher than 80% under this scenario.

In 2014, a revised power calculation was performed based on the results in the meta analysis by Bidard *et al* (1). Assumptions: Exponentially distributed survival times, groups of equal size, median PFS in the two groups of 11.4 and 6.5 months, respectively, an accrual period of 36 months and an extra follow-up period of 12 months after the last accrual. Then 154 patients would be sufficient to have 90% power to detect this difference in PFS as significant at the alpha level 0.05 with a log rank test.

The program PS: Power and Sample Size Calculation version 3.1.2, 2014 was used for these calculations (2).

References to supplementary information

1. Bidard FC, Peeters DJ, Fehm T, Nole F, Gisbert-Criado R, Mavroudis D, et al. Clinical validity of circulating tumour cells in patients with metastatic breast cancer: a pooled analysis of individual patient data. The lancet oncology 2014;15(4):406-14 doi 10.1016/s1470-2045(14)70069-5.
2. 2017/11/10. PS: Power and Sample Size Calculation. Department of Biostatistics, Vanderbilt University <http://biostat.mc.vanderbilt.edu/PowerSampleSize>. 2017/11/10.
