# Supplementary material for: Longitudinal enumeration and cluster evaluation of circulating tumor cells improve prognostication for patients with newly diagnosed metastatic breast cancer in a prospective observational trial
Source: Breast Cancer Res. 2018 Jun 8;20:48. doi: 10.1186/s13058-018-0976-0 (PMC5994056; doi:10.1186/s13058-018-0976-0)
Supplement: Supplementary file 2 — Figure S1. CTC count as a continuous variable in relation to presence of CTC clusters. (PDF 182 kb) [file 13058_2018_976_MOESM2_ESM.pdf]

Figure S1. CTC count as a continuous variable in relation to CTC-cluster presence at baseline, 1, 3 and 6 months.

Baseline

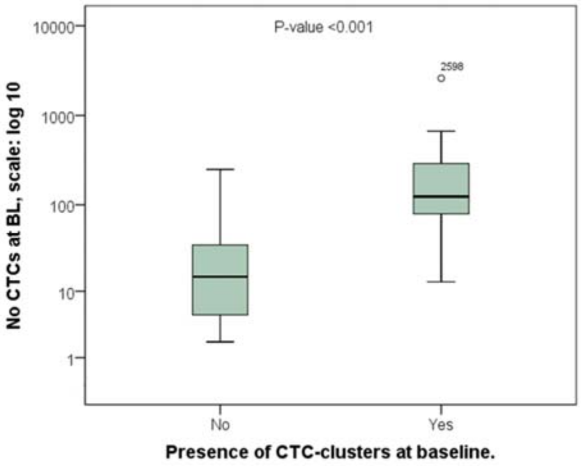

	CTC-cluster=No	CTC-cluster=Yes
**2-4 CTCs**	13	0
**5-19 CTCs**	23	2
**≥20 CTC**	26	28

1 month

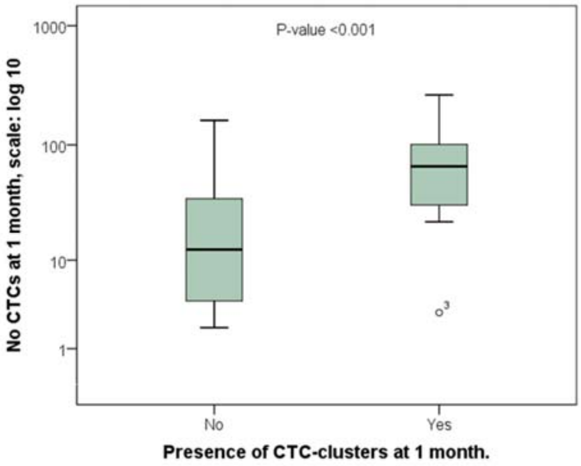

	CTC-cluster=No	CTC-cluster=Yes
**2-4 CTCs**	12	1
**5-19 CTCs**	12	0
**≥20 CTCs**	16	11

3 months

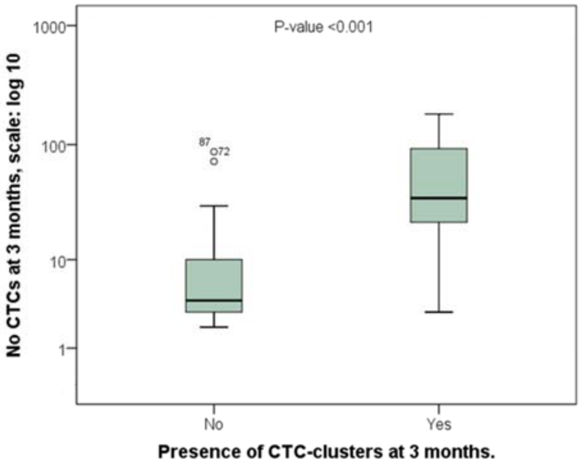

	CTC-cluster=No	CTC-cluster=Yes
**2-4 CTCs**	18	1
**5-19 CTCs**	11	2
**≥20 CTCs**	5	8

6 months

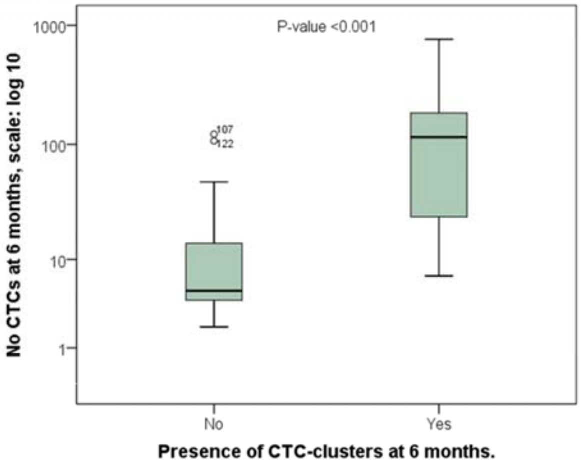

	CTC-cluster=No	CTC-cluster=Yes
**2-4 CTCs**	8	0
**5-19 CTCs**	8	2
**≥20 CTC**	5	8
