# Supplementary material for: Longitudinal enumeration and cluster evaluation of circulating tumor cells improve prognostication for patients with newly diagnosed metastatic breast cancer in a prospective observational trial
Source: Breast Cancer Res. 2018 Jun 8;20:48. doi: 10.1186/s13058-018-0976-0 (PMC5994056; doi:10.1186/s13058-018-0976-0)
Supplement: Supplementary file 3 — Table S1. Multivariable Cox regression analysis of prognostic variables. (PDF 426 kb) [file 13058_2018_976_MOESM3_ESM.pdf]

Table S1. Multivariable Cox regression analysis of prognostic variables included in the clinicopathological model

	PFS		OS	
	**HR (95% CI)**	***P*-value**	**HR (95% CI)**	***P*-value**
**Age at diagnosis MBC[a]**				
<65	1.00		1.00	
≥65	0.77 (0.49-1.23)	0.28	1.13 (0.65-1.96)	0.66
**ECOG**				
Ordinal scale (0, 1, 2)	1.02 (0.74-1.43)	0.89	1.48 (1.01-2.18)	0.05
**NHG**				
I-II	1.00		1.00	
III	1.19 (0.73-1.94)	0.48	0.98 (0.53-1.81)	0.95
**Subtype**				
HR+HER2-	1.00		1.00	
HER2+	0.74 (0.35-1.59)	0.44	0.94 (0.31-2.78)	0.90
HR-HER2-	2.48 (1.34-4.60)	0.004	4.99 (2.33-10.71)	<0.001
**Metastasis-free interval (years)**				
0	0.41 (0.13-1.26)	0.12	0.16 (0.02-1.33)	0.09
>0-3	1.00		1.00	
>3	0.77 (0.44-1.33)	0.35	0.71 (0.38-1.36)	0.31
**No. metastatic sites**				
<3	1.00		1.00	
≥3	1.55 (0.92-2.63)	0.10	2.07 (1.11-3.86)	0.02
**Site of metastasis**				
Non-visceral	1.00		1.00	
Visceral	1.82 (1.10-3.00)	0.02	1.65 (0.88-3.09)	0.12

[a]Cut off 65 years was chosen because it was the median age at diagnosis of MBC in this cohort of patients

Abbreviations: PFS, progression-free survival; OS, overall survival; HR, hazard ratio; MBC, metastatic breast cancer; ECOG, Eastern Cooperative Oncology Group; NHG, Nottingham histological grade; HR, hormone receptor; HER2, human epidermal growth factor receptor 2
