# Supplementary material for: Longitudinal enumeration and cluster evaluation of circulating tumor cells improve prognostication for patients with newly diagnosed metastatic breast cancer in a prospective observational trial
Source: Breast Cancer Res. 2018 Jun 8;20:48. doi: 10.1186/s13058-018-0976-0 (PMC5994056; doi:10.1186/s13058-018-0976-0)
Supplement: Supplementary file 4 — Table S2. Unadjusted Cox regression HRs for CTC count ≥ 5 vs < 5 CTCs, and CTC count ≥ 20 vs < 20 CTCs. (PDF 114 kb) [file 13058_2018_976_MOESM4_ESM.pdf]

Table S2. Unadjusted Cox regression HRs for CTC count ≥5 vs <5 CTCs, and CTC count ≥20 vs <20 CTCs baseline and during 1st line systemic therapy

	**PFS**	***P*-value**	**OS**	***P*-value**
**Baseline**				
≥5 vs <5 CTCs	1.75 (1.19-2.57)	0.004	2.55 (1.54-4.22)	<0.001
≥20 vs <20 CTCs	2.17 (1.49-3.16)	<0.001	3.35 (2.10-5.33)	<0.001
**1 month**				
≥5 vs <5 CTCs	2.11 (1.38-3.24)	0.001	4.24 (2.49-7.20)	<0.001
≥20 vs <20 CTCs	3.23 (2.04-5.10)	<0.001	6.20 (3.61-10.67)	<0.001
**3 months**				
≥5 vs <5 CTCs	2.08 (1.11-3.93)	0.02	3.10 (1.61-6.00)	0.001
≥20 vs <20 CTCs	8.66 (3.77-19.91)	<0.001	6.67 (3.24-13.74)	<0.001
**6 months**				
≥5 vs <5 CTCs	4.07 (1.94-8.51)	<0.001	8.58 (3.70-19.9)	<0.001
≥20 vs <20 CTCs	9.05 (3.35-24.42)	<0.001	17.53 (7.07-43.48)	<0.001
