# Supplementary material for: Longitudinal enumeration and cluster evaluation of circulating tumor cells improve prognostication for patients with newly diagnosed metastatic breast cancer in a prospective observational trial
Source: Breast Cancer Res. 2018 Jun 8;20:48. doi: 10.1186/s13058-018-0976-0 (PMC5994056; doi:10.1186/s13058-018-0976-0)
Supplement: Supplementary file 5 — Table S3. Change in CTC count in relation to progression versus non-progression at first radiological evaluation. (PDF 131 kb) [file 13058_2018_976_MOESM5_ESM.pdf]

Table S3. Change in CTC count from baseline to 1 month, and from BL to 3 months, in relation to progression *vs* non-progression at 1st radiologic evaluation performed at 3 months

	**No of patients**	**Non-PD**	**PD**	**OR (95% CI)**	***P*-value**
**Baseline CTC, 1 months CTC**					
<5 CTC, <5 CTC	65	57	8	1.00	
≥5 CTC, <5 CTC	33	28	5	1.27 (0.38-4.25)	0.70
<5 CTC, ≥5 CTC	0				
≥5 CTC, ≥5 CTC	35	22	13	4.21 (1.54-11.55)	0.005
**Baseline CTC, 3 months CTC**					
<5 CTC, <5 CTC	61	57	4	1.00	
≥5 CTC, <5 CTC	45	36	9	3.56 (1.02-12.43)	0.05
<5 CTC, ≥5 CTC	2	1	1	14.25 (0.75-272.64)	0.08
≥5 CTC, ≥5 CTC	23	14	9	9.16 (2.46-34.12)	0.001

Abbreviations: PD, progressive disease; CI, confidence interval; CTC, circulating tumor cell
