# Supplementary material for: Longitudinal enumeration and cluster evaluation of circulating tumor cells improve prognostication for patients with newly diagnosed metastatic breast cancer in a prospective observational trial
Source: Breast Cancer Res. 2018 Jun 8;20:48. doi: 10.1186/s13058-018-0976-0 (PMC5994056; doi:10.1186/s13058-018-0976-0)
Supplement: Supplementary file 6 — Table S4. Unadjusted Cox regression analyses of patient and tumor characteristics at baseline. (PDF 146 kb) [file 13058_2018_976_MOESM6_ESM.pdf]

Table S4. Unadjusted Cox regression analyses of patient and tumor characteristics at baseline

	PFS		OS	
	HR (95% CI)	*P*-value	HR (95% CI)	*P*-value
**Age at diagnosis MBC (years)**				
<65	1.00		1.00	
≥65	0.82 (0.57-1.17)	0.27	1.12 (0.73-1.73)	0.60
**ECOG**				
0	1.00		1.00	
1	1.27 (0.83-1.95)	0.27	1.68 (0.99-2.85)	0.05
2	1.34 (0.79-2.28)	0.28	2.67 (1.49-4.78)	0.001
**NHG**				
I-II	1.00		1.00	
III	1.37 (0.90-2.07)	0.14	1.37 (0.83-2.25)	0.22
**PT Tumor size (mm)**				
≤20 (T1)	1.00		1.00	
>20 (T2-4)	0.93 (0.63-1.36)	0.70	1.07 (0.68-1.70)	0.76
**PT Node status**				
Negative	1.00		1.00	
Positive	1.02 (0.63-1.65)	0.94	0.98 (0.65-1.48)	0.94
**Subtype**				
HR+HER2-	1.00		1.00	
HER2+	0.76 (0.43-1.32)	0.33	0.72 (0.34-1.52)	0.39
HR-HER2-	2.58 (1.58-4.20)	<0.001	5.42 (3.08-9.55)	<0.001
**Metastasis-free interval (years)**				
0	0.51 (0.29-0.92)	0.02	0.27 (0.14-0.55)	<0.001
>0-3	1.00		1.00	
>3	0.65 (0.40-1.04)	0.07	0.46 (0.27-0.77)	0.003
**No metastatic sites**				
<3	1.00		1.00	
≥3	2.07 (1.41-3.03)	<0.001	2.18 (1.41-3.37)	<0.001
**Site of metastasis**				
Non-visceral	1.00		1.00	
Visceral	1.69 (1.16-2.45)	0.006	1.59 (1.02-2.48)	0.04
**Baseline CA15-3 (kE/L)**				
<30	1.00		1.00	
≥30	1.40 (0.86-2.29)	0.17	1.56 (0.86-2.85)	0.15
**1st line treatment for MBC**				

Endocrine	1.00		1.00	
Chemotherapy	1.65 (1.10-2.47)	0.02	2.07 (1.25-3.42)	0.005
HER2-targeted	0.82 (0.42-1.61)	0.56	0.91 (0.37-2.23)	0.83

Abbreviations: PFS, progression-free survival; OS, overall survival; HR, hazard ratio; MBC, metastatic breast cancer; NHG, Nottingham histological grade; PT, primary tumor; HR, hormone receptor; HER2, human epidermal growth factor receptor 2
